# Exposure to Indoor Ferromagnetic Particulate Matter Monitored by Strawberry Plants and the Occurrence of Acute Respiratory Events in Adults

**DOI:** 10.3390/ijerph16234823

**Published:** 2019-11-30

**Authors:** Lieve Van Dyck, Hayat Bentouhami, Kyra Koch, Roeland Samson, Joost Weyler

**Affiliations:** 1 Social Epidemiology and Health Policy (SEHPO), University of Antwerp, Universiteitsplein 1, 2610 Wilrijk, Belgium; Hayat.Bentouhami@uantwerpen.be (H.B.); Joost.Weyler@uantwerp.be (J.W.); 2 Department of Bioscience Engineering, University of Antwerp, Groenenborgerlaan 171, 2020 Antwerpen, Belgium; Kyra.Koch@uantwerpen.be (K.K.); Roeland.Samson@uantwerpen.be (R.S.)

**Keywords:** indoor air pollution, exposure assessment, respiratory, ferromagnetic, environmental, epidemiology

## Abstract

Exposure assessment of air pollution in epidemiologic research remains a challenge. Previous studies showed that magnetic monitoring of strawberry leaves, based on Saturation Isothermal Remnant Magnetization (SIRM), is a valid tool for estimating the concentration of ambient particulate matter (PM). This study uses this assessment method for the first time in epidemiologic research to quantify indoor exposure to PM. In a nested case control study, we evaluated the association between ‘waking up by cough’ and indoor air pollution measured by SIRM of dust deposition on leaves of strawberry plants located in the bedroom in the general adult population. A multiple logistic regression was used to estimate the association between ‘waking up by cough’ and exposure to ferromagnetic particles of PM controlling for age, gender and smoking status. A cut-off of 10 µA was decided to define exposure status (high versus low). Using logistic regression, a crude odds ratio (OR) of 1.80 (95% CI: 0.90–3.60) for ‘waking up by cough’ was found. This association remained approximately the same after controlling for age, gender and smoking status (adjusted OR: 1.76; 95% CI: 0.60–5.30). We found an association between exposure to ferromagnetic particles and ‘waking up by cough’ in adults; however, it was not statistically significant. This environmental exposure assessment method could be a valuable alternative for expensive personal exposure measurement devices.

## 1. Introduction

Health effects of ambient air pollution are well documented by epidemiologic research [1,2,3]. In contrast, health effects of indoor air pollution are not consistently supported by scientific evidence [4,5].

However, people in industrialized countries spend 90% of their time in confined environments, where the concentration of pollutants may be two-to five-fold higher than outdoors [4]. These pollutants stem from specific indoor sources like environmental tobacco smoke (ETS), cooking and heating appliances among others [2,6]. Outdoor-indoor transfer of e.g., traffic-related air pollution (TRAP) also contributes to indoor air pollution. 

Effects of TRAP have been studied extensively [7,8] and strong evidence exists that particulate matter (PM) derived from road traffic adversely affects respiratory health, ranging from mild symptoms to severe pathologies [1,9]. However, it is still unclear which specific components of PM are responsible for this health effect [1]. PM derived from road traffic contains ferromagnetic components due to combustion processes or metallic wear and abrasion [10]. Toxicological studies have pointed to combustion-derived particles as having a high toxic potential. However, it has not been possible yet to quantify the contribution of different PM components to the effects on health [1].

TRAP is often measured using central site monitoring stations assessing the concentration of PM in a geographical area. The extent to which this ambient PM contaminates the indoor environment is not clear. A limitation of prior studies on indoor air pollution is that exposure assessment often has been based on surveying presence of indoor sources of air pollution [11]. Surveying the amount of cars or trucks that pass in front of one’s residence is also a crude method for assessing infiltrated pollution from traffic.

Attempts have been made to quantitatively assess indoor exposure to PM [12,13]. However, air sampling devices used indoors have some major drawbacks [14]. Also, personal monitors for PM are expensive and therefore cannot be used on a large scale and for a long time period. Alternative assessment methods of ambient air pollution are increasingly being used in place of central-site measurements [15]. Kardel et al. [16] applied a biomagnetic technique, Saturated Isothermal Remnant Magnetization (SIRM), in estimating the concentration of PM in ambient environment. The SIRM signal found on particular plant leaves depends on the concentration of ferromagnetic particles and therefore can be considered as a proxy for the concentration of ferromagnetic particles in the air [17]. In Antwerp, the SIRM signal on strawberry leaves was used to monitor ambient ferromagnetic PM [18]. In this Airbezen-project, the outdoor level of ferromagnetic PM varied between 10 and more than 400 µA over more than 700 points spread over the city of Antwerp and neighboring communities. Because of this prior experience in using strawberry plants as monitoring stations outdoors, we decided to use strawberry plants in our study to assess indoor exposure to ferromagnetic PM.

The main purpose of this study is to investigate the association between exposure to indoor ferromagnetic PM concentration, measured quantitatively using SIRM, and respiratory symptoms in the general adult population. Additionally, we aim to investigate the feasibility of this new assessment method in epidemiologic research. Finally, we investigate possible sources of indoor ferromagnetic PM by estimating associations between SIRM values and reported PM sources.

## 2. Materials and Methods 

We conducted a nested case-control study to investigate the effect of ferromagnetic PM concentration in the bedroom on a nocturnal respiratory event. We chose the bedroom as location of exposure since it is the most confined room in the house where people spend on average 7 to 8 h ceaseless. This also implies that we investigated acute effects of exposure, not taking total indoor exposure or history of exposure into account. Acute effects are mostly mild symptoms which can be a healthy reaction to irritation of airways but nonetheless can have a profound effect on people’s quality of sleep and activity level. Since coughing is the most common respiratory event in the general population and most likely to be evoked by exposure to PM concentration, we chose ‘waking up by cough during the past 12 months’ as case definition.

The study was conducted in accordance with the Declaration of Helsinki Principles. Ethical approval was obtained in the ECRHS study for each center from the appropriate institutional ethics committee (ethical approval code 11/41/288-UA). All subjects participating in this study provided informed consent.

### 2.1. Study Population 

Subjects who participated in 2013 in the European Community Respiratory Health Survey (ECRHS III) [19,20,21] and were living in the two participating Antwerp centers were eligible as study participants. ECRHS III is the second follow-up of the ECRHS, a longitudinal respiratory cohort study initiated in 1990. In these two Antwerp centers, 364 subjects were enrolled in ECRHS III. For practical reasons, the selection of participants was limited to subjects living in Antwerp and in a radius of 30 km from Antwerp. After restriction by residence and traceability, 294 subjects were approached for their approval to assess their exposure to indoor air pollution. The consent rate was very high (98%). After exclusion by several other factors (vacation, forgetfulness of participants), 223 subjects (75%) could be enrolled in our study and a strawberry plant was delivered to their home address. Finally, 193 participants responded (response rate 86%) either by sending us an envelope with a sample of strawberry leaves and/or by reporting that their plant had withered.

From the ECRHS III, three questions concerning nocturnal respiratory events were used to define our cases and controls.

1. Have you at any time during the past 12 months woken up with a feeling of chest tightness?
2. Have you at any time during the past 12 months woken up by an attack of shortness of breath?
3. Have you at any time during the past 12 months woken up by a cough?

Cases are those reporting at least ‘waking up by cough’ as a nocturnal respiratory event during a 1-year time period from 2012 to 2013. Controls are those reporting not been woken up by any respiratory event during the same time period, so neither by cough, shortness of breath nor chest pain.

### 2.2. Exposure Assessment

Assessment of indoor ferromagnetic PM concentration was carried out using strawberry plants as monitoring stations. Strawberry plants were located in the bedroom during 2 months in order to determine the SIRM signal of accumulated dust deposition on strawberry leaves. Distribution among participants was carried out during March and April 2015. This time period was chosen because plants grow well during this period. 

For practical reasons, two different methods were used for dispatching the strawberry plants among participants. One-hundred-and-twenty-six strawberry plants were delivered by car to the subject’s home address. For 97 subjects living in densely urban environment, we provided two collection points where they could fetch their plant. 

At the end of the measurement period, participants needed to sample five full-grown leaves. Sampling was done according to a strict protocol, only touching the petiole and sending the leaves in a paper envelope the same day for analysis to the laboratory of Environmental and Urban Ecology of the University of Antwerp (for detailed information, see the Supplementary Data: File S1).

Of each sample, leaf area was determined using a Li-3100 leaf area meter (LI-COR Environmental^®^, LI-COR biosciences, Lincol, NE, USA). Samples were packed in cling film for immobilization and pressed in a 10 cm^3^ plastic container. Following the protocol of Matzka and Maher, 1999 and Kardel et al., 2011, these containers were magnetized with a pulsed magnetic field of 1T using a Molspin pulse magnetizer (Molspin Ltd.^®^, Oxfordshire, UK). Subsequently the SIRM signal was determined using a Molspin Minispin magnetometer (Molspin Ltd., Oxfordshire, UK) with high sensitivity (−0.1 × 10^−10^ A/m^2^). After every 10 measurements, the magnetometer was calibrated using a magnetically stable rock specimen, as described by Mitchell et al., 2010. Each sample was measured twice to minimize measurement errors. If the difference between two measurements was more than or equal to 1 µA, measurements were repeated. Each SIRM value is the average of two measurements on the same sample. Measured SIRM values were then normalized for sampling pot volume (10 cm^3^) and leaf area (cm^2^) which leads to a leaf area normalized SIRM-value expressed as A in the following formula.

(1) $$SIRM\left(A\right)\;=\;\frac{Intensity\left(\frac{mA}{m}\right)*Volume\;box\;\left(m^{3}\right)}{Surface\;leaf\;\left(m^{2}\right)}$$

The actual measurement period was assessed by subtracting the date the envelope was sent for analysis from the date of dispatch. In case the date on the envelope was not readable (22%), the date of analysis was used. Analysis was carried out within 5 days after reception of the sample. SIRM values were standardized by mean measurement period (formula: SIRM value/length of the actual measurement period × 60 days) as there was variability in actual measurement periods. This was due to forgetfulness of participants of the instructed sampling date or to the fact that participants decided to sample earlier due to withering of the plant.

### 2.3. Statistical Analysis

SIRM values were categorized in four arbitrarily chosen categories (0–5, 5–10, 10–15, >15 µA). To decide on the nature of the association, relative frequencies of ‘waking up by cough’ were calculated for these four categories (Table 1). Distributions of other relevant characteristics are presented for cases and controls together with an OR, 95% CI and p-values, using bivariate logistic regression (Table 2). To investigate the association between dichotomized SIRM and ‘waking up by cough’, we performed multiple logistic regression adjusting for age, gender and smoking status (Table 3). Respiratory illnesses, respiratory medication use and smoking status were considered potential effect-modifiers as they might be part of the causal chain. Associations between possible sources of SIRM and (continuous) SIRM values (n = 162) were estimated using Kruskal-Wallis test and Mann-Whitney U test when appropriate (Appendix A). Statistical analyses were conducted in R.

## 3. Results

### 3.1. Study Population and SIRM Values

SIRM values were determined on 162 samples of leaves, which is 73% of the distributed strawberry plants. Reasons why SIRM could not be determined include not receiving a sample due to the plant’s withering, excessive withering of leaves or presence of greenfly. Still, 14 SIRM values could be obtained from moderately withered leaves and 2 from leaves with greenfly. The distribution of SIRM values is right skewed with a median SIRM value of 8.71 µA. There was considerable variability in SIRM values (Inter Quartile Range (IQR) 11.81 µA) with a minimum value of 0.85 µA and maximum value of 50.95 µA. Mean measurement period was 60 days (SD 10), with a minimum of 35 and a maximum of 122 days.

### 3.2. Cases and Controls

We applied our case definition on 162 subjects for whom we could determine a SIRM value. Forty-nine subjects reported being woken up by cough, of which one subject also reported being woken up by an attack of shortness of breath and three subjects also reported being woken up by a feeling of chest tightness. Four of the 49 subjects experienced all three symptoms. One-hundred-and-one subjects did not experience any nocturnal symptoms and were selected as controls. Twelve subjects experienced only waking up by chest tightness or shortness of breath but did not experience waking up by cough. These were not included as cases.

### 3.3. Defining Exposure Status

We decided to study exposure in a dichotomous scale instead of a continuous scale because we could not assume a linear relationship in the logit between SIRM and ‘waking up by cough’. Based on the distribution of cases and controls across SIRM categories, we chose 10 µA as the critical threshold leading to more or less balanced data: 41.3% subjects were exposed to SIRM values above 10 µA and 58.7% subjects were exposed to SIRM values below 10 µA.

### 3.4. Relevant Characteristics of Cases and Controls

We found a significant relationship between respiratory illnesses and waking up by cough’ (*p* = 0.007) and between inhalation medication and ‘waking up by cough’ (*p* = 0.033).

### 3.5. Association Between Dichotomized SIRM and ‘Waking Up By Cough’

The observed crude OR was 1.80 (95% CI: 0.90–3.60), χ^2^ = 2.254, *p* = 0.093. Participants exposed to more than 10 µA SIRM had 1.8 times higher odds for ‘waking up by a cough’ than those exposed to less than 10 µA SIRM, although this association was not statistically significant at the 0.05 level (*p* = 0.093). After adjusting for age, gender and smoking status in multiple logistic regression, the OR was 1.76 (95% CI: 0.60–5.30).

Besides respiratory illness, we also considered smoking status, ETS and respiratory medication use as potential effect-modifiers, but we found no statistical evidence for modification of the association (interaction term p values resp. *p* = 0.926, *p* = 0.662, *p* = 0.907, *p* = 0.251).

### 3.6. Associations Between Presence of PM Sources and SIRM Values

A significant positive association was found between continuous SIRM values and reported amount of cars, but not with the reported amount of heavy trucks that pass in front of someone’s residence (Appendix A). There were no significant associations between SIRM values and other (investigated) possible PM sources (Appendix A).

## 4. Discussion

Our results suggest an association, although it is not statistically significant, between exposure to ferromagnetic particles and the occurrence of ‘waking up by cough’ in adults.

The association we observed between SIRM and ‘waking up by cough’ hardly changed after adjustment for age, gender and smoking status (OR_crude_ = 1.80 (95% CI: 0.90–3.60); OR_adjusted_ = 1.76 (95% CI: 0.60–5.30). Including other potential confounders did not further change the association. We could not control for other respiratory infections which could have led to misclassification bias. However, we do not think this possible misclassification would be differential. It is also possible that ‘waking up by cough’ was evoked by other factors like indoor temperature or other components of air pollution that co-occur with ferromagnetic particles like nitrogen oxide (NOx). Since we did not have data concerning these factors or other environmental pollutants, we could not take these into account. 

We obtained a relatively large adjusted odds ratio (OR = 1.76) in contrast to earlier reported acute respiratory effects with relative frequencies of 1.005–1.010 [2]. The first (obvious) explanation is the fact that we decided to dichotomize the exposure variable, while in previous studies exposure was studied on a continuous scale with some arbitrarily chosen unit. Secondly, the obtained OR can also be a consequence of our choice of ‘waking up by cough’ as a health outcome of interest as it is not so rare in the adult population. The reported acute effects with relative frequencies of 1.005–1.010 are particularly small (but highly significant and relevant) due to multifactorial causes of respiratory symptoms. Likewise, an OR of 1.76 is not very large for an association between a dichotomized exposure variable and a frequently occurring health outcome.

Selection bias is possible because people living in dense urban environments were asked to fetch their plant at a collection point. It can be suspected that those who experience more symptoms or live in a more (perceived) polluted urban environment (Antwerp City) are more inclined to make an effort to fetch their plant. 

We tried to minimize measurement error by measuring each sample twice. However, it is possible that measurement bias is an issue, since we included dried leaves from withered plants or plants that got greenfly in our analysis. It is unknown whether leaves that withered or got greenfly give raise to significantly different SIRM values than healthy leaves. Therefore, statistical analysis was repeated without SIRM values of leaves with greenfly and without SIRM values of withered leaves which could have led to exposure misclassification. This resulted in a crude OR of resp. 1.85 (95% CI: 0.92–3.71) and 1.43 (95% CI: 0.68–3.00). We do not know however whether withered leaves capture ferromagnetic particles differently and therefore produce biased measurements.

Our association is based on several assumptions about exposure assessment. 

Assessment of respiratory outcomes in ECRHS III happened in 2013, 2 years before our exposure estimation in 2015. We therefore assume that the subjects’ indoor exposure status to PM has not substantially changed over the past 2 years and that assessment in 2015 is a good proxy of their exposure to PM in 2013. In order to allow for this assumption, we selected only study participants who still lived in the same house in 2015 as in 2013. We did not take into account how long they lived there since we are not investigating the effects of chronic exposure. Because of the nature of our study design, the directionality of the association is not guaranteed. We do not suspect, however, that subjects were moved because of their nocturnal complaints as they do not know their true exposure status. 

Another assumption of our study is that exposure in the bedroom causes acute effects of ‘waking up by cough’ within hours. Because of a lack of data, we could not standardize for exposure time in the bedroom. We also do not know whether ‘waking up by cough’ is a delayed effect from peak exposure or totality of exposures during the day.

Our findings also seem to support the notion of absence of a lower limit of air pollution under which no health-related effects occur, since we have shown that risk of ‘waking up by cough’ increases if you are exposed to PM concentrations as low as the urban ambient background level of 10 µA, as was derived from the AIRbezen project. 

Finally, we looked at the association between SIRM and possible PM sources in order to verify whether reducing exposure to indoor ferromagnetic PM concentration in an intervention study would be feasible.

We found only a significant association between SIRM and car passage. This suggests that indoor PM concentrations most likely originate from TRAP that infiltrates indoors. Measures such as opening windows occur only outside rush hour times and, therefore, may help to minimize indoor air pollution. We did however not find a statistically significant association between SIRM and heavy truck passage. This can be explained by several factors that mediate infiltration of air from outdoor to indoor. Besides bedroom floor and distance of the bedroom from the roadside, also ventilation determines outdoor-indoor transfer. It is e.g., conceivable that people who report a lot of trucks passing in front of one’s residence are less inclined to open their windows for ventilation. Even though a lot of trucks pass, still PM concentrations could differ considering on what floor the apartment is situated. Since we found no convincing evidence for the existence of indoor PM pollution sources in the bedroom, the reduction of air pollution on an individual level seems rather limited.

This measurement method is promising to monitor concentrations after interventions and might be applied in clinical populations in order to investigate whether lowered ferromagnetic PM concentration would reduce their respiratory symptoms.

## 5. Conclusions

Monitoring PM concentration using strawberry plants is a promising tool for the assessment of indoor exposure to specific PM constituents relevant for health outcomes. The first strength of this assessment method is its low cost. This implies that this method can be used on a large scale and for a relatively long measurement period. Another strength of this assessment method is that the nature of the method contributes to a high participation rate.

In future research, we suggest optimizing this new measurement method by investigating whether other indoor ornamental plants are more suited to assess indoor exposure to ferromagnetic PM. This method can be implemented in multiple exposure estimation approaches. Using monitoring of strawberry plants together with other quantitative measurement devices may help to reduce exposure error and make comparisons between different studies possible. By measuring SIRM on samples collected from the inside as well as on the outside of a window, differences between indoor and outdoor ferromagnetic PM concentrations can be determined. We suggest using this new approach on a larger scale to increase statistical power and to validate our observed association between acute respiratory events and exposure to indoor ferromagnetic PM concentration. 

## Figures and Tables

**Table 1:** Distribution of cases and controls in different SIRM categories. SIRM: Saturation Isothermal Remnant Magnetization.

SIRM Exposure	Cases (n = 49)	Controls (n = 101)	0R (95% CI)	*P*-value
	n (%)	n (%)		
0–5 µA	7 (14.3%)	25 (24.7%)	(ref)	
5–10 µA	17 (34.7%)	39 (38.6%)	1.56 (0.58–4.51)	
10–15 µA	8 (16.3%)	12 (11.9%)	2.38 (0.70–8.36)	
>15 µA	17 (34.7%)	25 (24.7%)	2.43 (0.88–7.23)	

**Table 2:** Socio-demographic characteristics and respiratory risk factors of cases and controls (n = 150, ECRHS III, 2013).

Characteristics and Respiratory Risk Factors	Cases	Controls	OR (95% CI)	*P*-value
	(n = 49)	(n = 101)		
Age				
Mean (SD)	54.9 (6.51)	54.3 (7.04)	1.013 (0.96–1.06)	0.607
Min-max	42–66	42–67		
Gender				
Male (1)	17 (35%)	45 (45%)		
Female (0)	32 (65%)	56 (55%)	0.66 (0.32–1.33)	0.33
Smoking Status				
Never smoked	29 (60%)	51 (50%)	(ref)	
Ex-smoker	13 (26%)	36 (36%)	0.63 (0.28–1.37)	0.254
Current smoker	7 (14%)	13 (13%)	0.95 (0.32–2.59)	0.917
Environmental Tobacco Smoke (ETS)				
Not exposed	36 (73%)	83 (82%)		
Exposed	12 (24%)	17 (17%)	1.63 (0.70–3.74)	0.354
Bedroom				
No carpet	28 (57%)	70 (69%)		
Carpet	19 (39%)	29 (29%)	1.64 (0.79–3.39)	0.183
No radiator	11 (22%)	21 (21%)		
Radiator	35 (71%)	77 (76%)	0.87 (0.38–2.05)	0.738
No moisture or mold	46 (94%)	93 (92%)		
Moisture and/or mold	3 (6%)	8 (8%)	0.76 (0.16–2.76)	0.693
No pets	43 (88%)	90 (89%)		
Cat and/or dog	6 (12%)	11 (11%)	1.14 (0.37–3.21)	0.806
Heating appliance				
No closed woodstove	28 (82%)	64 (89%)		
Closed woodstove	6 (18%)	8 (11%)	1.71 (0.52–5.40)	0.357
Respiratory illnesses **				
No respiratory illnesses	34 (69%)	89 (88%)		
Respiratory illnesses	15 (31%)	12 (12%)	3.27 (1.40–7.84)	0.007
Respiratory Medication (past year)				
No inhalation medication	41 (84%)	97 (96%)		
Inhalation medication	6 (12%)	3 (3%)	4.73 (1.19–23.28)	0.033
No oral medication *	42 (86%)	94 (93%)		
Oral medication	6 (12%)	6 (6%)	2.24 (0.66–7.55)	0.184
No medication	40 (82%)	92 (91%)	2.30 (0.84–6.38)	0.101
Total medication	9 (18%)	9 (9%)		

Coding: no exposure: 0; exposure: 1; * medication for respiratory illness, ** asthma, chronic bronchitis, bronchial hyperresponsiveness, bronchitis, incipient pneumonia, pertussis, pneumothorax, pneumonia, pleurisy, primo infection, tuberculosis.

**Table 3:** Crude and adjusted OR for SIRM.

SIRM Exposure	Cases	Controls	OR (95%CI)	*P*-value
	(n = 49)	(n = 101)		
0–10 µA	24	64	1.80 (0.90–3.60)	0.093
10 + µA	25	37	1.76 * (0.60–5.30)	0.305

* OR adjusted for age, gender and smoking status (current smoker).

## Supplementary Materials

The following are available online at https://www.mdpi.com/1660-4601/16/23/4823/s1, File S1: AIRBEZEN protocol.

## Appendix A

**Table A1:** Association between SIRM and possible PM sources. * Kruskal-Wallis Test ** Mann-Whitney U Test.

	SIRM (median) µA	*P*-value
Car passage		0.035 *
Less than 5 per hour	6.57	
Between 5 and 20 per hour	8.22	
Between 21 and 80 per hour	10.94	
More than 80 per hour	9.73	
Heavy trucks passage		0.079 *
Less than 5 per hour	8.07	
Between 5 and 20 per hour	10.94	
Between 21 and 80 per hour	16.8	
More than 80 per hour	7.52	
Bedroom floor		0.940 **
Ground floor	9	
Second floor	7.95	
Exposure to ETS	9.36	0.365 **
No exposure to ETS	8.48	
Cooking appliances		0.212 **
Gas cooker	9.73	
Electricity	7.94	
Heating appliances		0.279 **
Closed woodstove	10.22	
No closed woodstove	8.64	
Carpets		0.871 **
Carpets	8.28	
No carpets	8.56	

**Table A2:** Logistic Regression Analysis for the association between SIRM and ‘waking up by cough’, adjusted for age, gender and smoking status.

Predictor	β	Seβ	Wald’s χ^2^	*p*	*e*^β^ (*Odds Ratio*)
Constant	−4.347	2.318	3.518	0.061	0.013
SIRM	0.567	0.554	1.051	0.305	1.764
Gender	−0.175	0.565	0.096	0.757	0.840
Age	0.058	0.040	2.074	0.150	1.059
Smoking Status	0.504	0.607	0.688	0.407	1.655

Smoking status = current smoker.
