# Supplementary material for: Exposure to Indoor Ferromagnetic Particulate Matter Monitored by Strawberry Plants and the Occurrence of Acute Respiratory Events in Adults
Source: Int J Environ Res Public Health. 2019 Nov 30;16(23):4823. doi: 10.3390/ijerph16234823 (PMC6926724; doi:10.3390/ijerph16234823)
Supplement: Supplementary file 1 [file ijerph-16-04823-s001.pdf]

AIRBEZEN protocol

Dear volunteer,

Thank you for participating in the follow-up study of the Airbezen project.

In this study we want to investigate the association between respiratory complaints and air quality in the bedroom. The air quality is measured using a strawberry plant.

We want to give you some instructions regarding the care of the strawberry plant and the plucking weekend.

1. Place the plant indoors in the bedroom for 2 months. Give the plant enough water but do not fertilize or replant it.

2. The leaves must be plucked after 2 months. Use the coded envelope to send the leaves.

   a. Pluck 5 (sets of) leaves, fully grown, large and undamaged. **Pluck them on the leaf stalk without touching the leaf.**
   b. Insert the leaves in the coded and stamped envelope
   c. Send the envelope (franking is not necessary)

You will receive a reminder email from us shortly before the plucking weekend.

Send the leaves within 48 hours after plucking. If you cannot post the leaves the same day, keep them in an open envelope in the fridge for as long as possible.

You can view the exact procedure in the following video:

https://www.uantwerpen.be/nl/onderzoeksgroep/endemic/onderzoek/projecten/airbezen/filmpjes-en-foto-s/

Thank you in advance for your cooperation!
